# Biomechanical modelling infers that collagen content within peripheral nerves is a greater indicator of axial Young’s modulus than structure

**DOI:** 10.1007/s10237-024-01911-w

**Published:** 2024-11-25

**Authors:** Eleanor A. Doman, Nicholas C. Ovenden, James B. Phillips, Rebecca J. Shipley

**Affiliations:** 1 Department of Mathematics, University College London, Gower St, London, WC1E 6BT UK; 2 Department of Mathematics, The University of Manchester, Oxford Road, Manchester, M13 9PL UK; 3 Department of Pharmacology, University College London, Brunswick Square, London, WC1N 1AX UK; 4 Department of Mechanical Engineering, University College London, Torrington Place, London, WC1E 7JE UK

**Keywords:** Peripheral nerves, Biomechanics, Model parametrisation, Multiscale modelling

## Abstract

The mechanical behaviour of peripheral nerves is known to vary between different nerves and nerve regions. As the field of nerve tissue engineering advances, it is vital that we understand the range of mechanical regimes future nerve implants must match to prevent failure. Data on the mechanical behaviour of human peripheral nerves are difficult to obtain due to the need to conduct mechanical testing shortly after removal from the body. In this work, we adapt a 3D multiscale biomechanical model, developed using asymptotic homogenisation, to mimic the micro- and macroscale structure of a peripheral nerve. This model is then parameterised using experimental data from rat peripheral nerves and used to investigate the effect of varying the collagen content, the fibril radius and number density, and the macroscale cross-sectional geometry of the peripheral nerve on the effective axial Young’s moduli of the whole nerve. Our results indicate that the total amount of collagen within a cross section has a greater effect on the axial Young’s moduli compared to other measures of structure. This suggests that the amount of collagen in a cross section of a peripheral nerve, which can be measured through histological and imaging techniques, is one of the key metrics that should be recorded in the future experimental studies on the biomechanical properties of peripheral nerves.

## Introduction

**Fig. 1:**
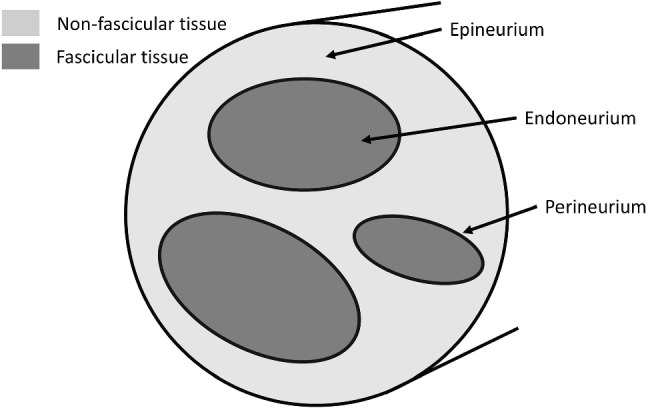
Diagram of nerve cross section with three fascicles with tissue compartments labelled

Peripheral nerves are complex hierarchical tissues that transmit information between the central nervous system and all other tissues in an organism, such as those in organs and muscles. They are primarily formed of three main cellular components: (i) nerve fibres, comprising of axons that conduct action potentials and Schwann cells that support and myelinate the axons; (ii) blood vessels, which supply oxygen and nutrients to the nerve; and (iii) an extracellular matrix (ECM) surrounding the nerve fibres and blood vessels, made up of collagen fibrils and other proteins providing structural support. Unusually for cells in biological tissues, the axons of peripheral neurons can be up to 1 m in length, (Sunderland 1968) and are approximately 1 to 20 mm in diameter, (Griffith and Guggenberger 2021). Therefore, understanding the mechanical properties of nerves that allow these long thin delicate cells to accommodate to the wide range of motions and stresses to which this tissue is exposed is an important area of study. Collagen fibrils, nerve fibres and blood vessels typically run parallel, along the length of the nerve. These aligned slender structures are primarily arranged within bundles called fascicles, the central section of which is known as the endoneurium, and are surrounded by a protective layer known as the perineurium and connective tissue known as the epineurium, as shown in Fig. 1. Human peripheral nerves typically contain between 3 and 42 fascicles, (Brill and Tyler 2017), while sciatic nerves in rats, the most commonly used animal model in peripheral nerve studies, have between 1 and 3 fascicles, (Phillips et al. 2004).

It is estimated that 2.8% of all trauma injuries include a peripheral nerve injury (PNI), (Noble et al. 1998). PNI can take a number of forms with differing degrees of severity; from stretching or compression, resulting in sections of the nerve that have axon and myelin damage, compromised mechanical support or insufficient blood supply, through to laceration type injuries, resulting in a break in the continuity of the nerve fibres. The classifications of types of injuries are extensively discussed in the wider literature including Burnett and Zager (2004), Lee and Wolfe (2000) and Deumens et al. (2010). While not all PNIs require surgical intervention, for those PNIs that result in a gap in the continuity of the nerve larger than 1 cm, the gold standard treatment is a nerve autograft. Here, a section of uninjured nerve is transplanted into the injury site to restore the continuity of the nerve and provide mechanical and chemical guidance to axons as they connect and regrow. However, it is estimated that less than 50 % of patients who undergo this surgical intervention regain function beneficial to their day-to-day lives (Lee and Wolfe 2000). New treatments for PNI are being developed, including the use of engineered replacement tissues to be implanted into the injury site, as discussed in Georgiou et al. (2013), Ahn et al. (2015), Chiono and Tonda-Turo (2015), Belanger et al. (2018) and Monfette et al. (2023). But for an engineered implant to be an effective treatment, the implant must match the mechanical properties of the original nerve to prevent further injury, (Wilcox et al. 2020). Data on the mechanical properties of human nerve tissue are scarce within the literature and difficult to obtain, (Giannessi et al. 2019; Barberio et al. 2019), and often are insufficient to examine the range of different nerve mechanics present within a population of different individuals, nerves and nerve sections. This sparsity of data is due to the requirement that the mechanical properties of biological tissues must be measured soon after extraction from the body to reduce the effect of degradation and the lack of available intact nerve tissue. However, histological and structural information, such as number and size of the fascicles, is significantly more accessible as these data can be obtained from fixed tissue.

Past experimental work has already investigated the question of whether this readily accessible structural information can be used to infer information on the mechanical properties of peripheral nerves. The vast majority of readily available human peripheral nerve tissue is diseased or damaged, and consequently, the majority of data on the normal function of peripheral nerves come from rat models. In Phillips et al. (2004), the local strain, stiffness, number of fascicles and proportion of connective tissue were measured in the joint and non-joint regions of rat sciatic and median nerves. It was seen that joint regions underwent a greater local strain during limb movements, suggesting joint regions to be less stiff than non-joint regions; however, no trend was found between the number of fascicles or the amount of connective tissue. Following on from this, Mason and Phillips (2011) investigated the collagen fibrils in the same selection of rat joint and non-joint regions. It was seen that thinner collagen fibrils with a greater density occurred in joint regions compared to non-joint regions, leading to the hypothesis that thinner fibrils with a higher density correlate to less stiff regions.

To design new engineered nerve replacement tissues, we need a robust methodology to establish the ideal mechanical ranges for implants into common peripheral nerve injury sites, in terms of commonly used mechanical engineering parameters for stiffness, such as Young’s modulus. In this paper, we construct a mathematical model to infer the effective Young’s modulus of a rat peripheral nerve based on experimental data from Phillips et al. (2004), Mason and Phillips (2011) and Liu et al. (2012) and examine which experimentally measured parameters have the greatest effect on the effective Young’s modulus. In Sect. 2, we extend an existing model for the mechanics of a fibre-supported structure from Doman et al. (2022) and parametrise this model using experimental data. We then examine, in Sect. 3, whether the model confirms the experimental hypothesis that thinner fibrils with a higher density corresponds to a less stiff nerve. Further analysis is then undertaken in Sect. 3 to examine which histological and structural parameters have the greatest effect on the Young’s modulus of the overall nerve before discussing the implications of our study in Sect. 4.

## Methods

In this section, we describe the mathematical model developed to replicate the mechanical properties of peripheral nerves. We hypothesise that the two key structural elements of the peripheral nerve that influence the effective mechanical properties are: (a) the density and arrangement of collagen fibrils throughout the nerve and (b) the arrangement of endoneurial and epineurial tissue within the nerve, which accounts for variation within a population of nerve sections. As shown in Mason and Phillips (2011), the arrangement of collagen fibrils within the endoneurium and epineurium is regular and relatively monodisperse. This allows for the assumption of microscale periodicity and the long thin nature of the peripheral nerve ensures a separation across length scales enabling the use of homogenised models. Here, the separation of length scales and periodicity is exploited to derive equations for the mechanical behaviour of the macroscale, in terms of the microscale structure and mechanical properties. The homogenised model for fibre-supported slender composites used in this paper is based on the approach in Doman et al. (2022). This model allows us to explore the impact of variation of the collagen fibril density and arrangement on the effective tissue mechanical properties within a single material layer, namely the endoneurium or epineurium. In this paper, we extend this model to a 2-layered structure, allowing us to explore variation due to both the endoneurium and epineurium layers on the mechanical properties of the whole nerve.

Once the model is extended, in Sect. 2.3 we describe the process of parameterising our 2-layer model to replicate the mechanical behaviour observed in Mason and Phillips (2011). First, we explore which parameters have the greatest effects on the range of predicted mechanical properties, before proceeding to parameterise the model based on the macroscale behaviour of the joint region of the rat sciatic nerve, as shown in Mason and Phillips (2011).

### Homogenisation of a composite fibre-supported material

Consider a long slender body, resembling a peripheral nerve, primarily made up of a material of type B, containing uniform fibres of another material (type A) that run longitudinally through the entire body. The two materials are characterised as a linearly elastic solids, assuming small strains within physiological conditions. The cross-sectional areas of the individual fibres are significantly smaller than the cross section of the nerve, enabling the application of asymptotic homogenisation to construct constitutive equations for the mechanical behaviour of the whole material. We assume the microscale is periodic and define a microscale cell around a singular fibre cross section which will fully tile the macroscale cross section. The area of material *j* (for $j = A$ or *B*) in a single cell is denoted $|\Omega _j|$ and the boundary between the two materials within the cell is denoted $\gamma$.

**Fig. 2:**
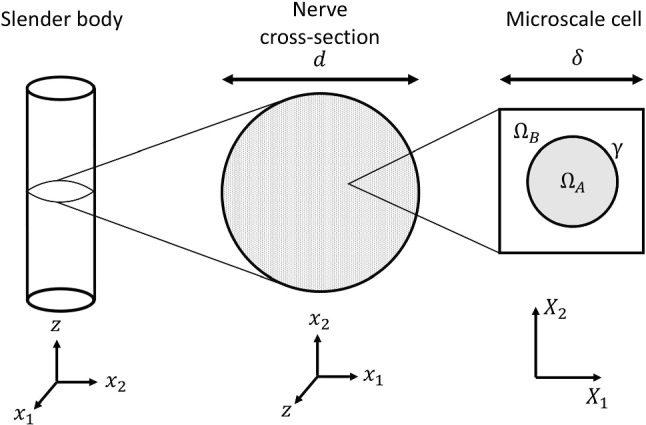
Slender body with cross-sectional diameter *d* containing material (type B) and fibres of material (type A). Example microscale cell around a single fibre cross section is demonstrated on right

A Cartesian coordinate system, $\textbf{x} = (x_1, x_2, z)^T$, is used, where *z* is the longitudinal direction of the slender body. We define the typical diameter of the cross section of the nerve to be *d* and the typical diameter of the collagen fibril to be $\delta$, as shown in Fig. 2. It is assumed that variations in the diameter of the collagen fibril are negligible. We denote the displacement vector within material *j* (for $j = A$ or *B*) as $\textbf{u}_j = (u_j, v_j, w_j)^T$ and take material *j* to have Lamé parameters $\lambda _j$ and $\mu _j$. Note that $u_A$, $u_B$, $v_A$ and $v_B$ are the displacements within the lateral cross section of the nerve, and therefore scale with $\delta$, whereas $w_A$ and $w_B$ are longitudinal displacements and so scale with *d*. To exploit the two scales, we introduce the Cartesian microscale coordinates in the cross-sectional reference frame of the fibril as $\textbf{X} = (X_1, X_2)^T$ such that $(x_1, x_2)^T = \epsilon \textbf{X}$ where $\epsilon$ is the non-dimensional length ratio $\frac{\delta }{d}$. We take $\epsilon$ to be small and expand all the non-dimensionalised displacements relative to this small parameter in the form of

1 $$\begin{aligned} f(\textbf{x}, \textbf{X})= f^{(0)}(\textbf{x}, \textbf{X})+\epsilon f^{(1)}(\textbf{x}, \textbf{X})+ \cdots \end{aligned}$$

As described in Doman et al. (2022), we are able to substitute these asymptotic expansions into the steady linearly elastic Navier equations for a periodic structure. The leading order displacements are seen to be independent of the microscale allowing us to simplify the displacement notation as

2 $$\begin{aligned}&u_A^{(0)}=u_B^{(0)} = u_1(\textbf{x}), \quad v_A^{(0)}=v_B^{(0)} = u_2(\textbf{x}) \nonumber \\&\quad \text {and} \quad w_A^{(0)}=w_B^{(0)} = w(\textbf{x}). \end{aligned}$$

The resultant effective macroscale equations are

3 $$\begin{aligned}&(K_{pqik}+K_{pqki}) \frac{\partial ^2 u_p}{\partial x_q \partial x_i}+(K_{ik}^0+K_{ki}^0) \frac{\partial ^2 w}{\partial z \partial x_i} \nonumber \\&\quad + H_{pk}\left( \frac{\partial ^2 w}{\partial x_p \partial z}+\frac{\partial ^2 u_p}{\partial z^2}\right) +G_{pq} \frac{\partial ^2 u_p}{\partial x_k \partial x_q} \nonumber \\&\quad +G^0 \frac{\partial w}{\partial x_p \partial z}+A_1 \left( \frac{\partial ^2 u_i}{\partial x_k \partial x_i}+\frac{\partial ^2 w}{\partial z \partial x_k}\right) \nonumber \\&\quad +A_2 \left( \frac{\partial ^2 u_k}{\partial x_i \partial x_i}+\frac{\partial ^2 u_k}{\partial z^2}\right) =0, \end{aligned}$$

and

4 $$\begin{aligned}&H_{pi} \left( \frac{\partial ^2 w}{\partial x_p \partial x_i}+\frac{\partial ^2 u_p}{\partial z \partial x_i}\right) +A_2 \frac{\partial ^2 w}{\partial x_i \partial x_i}\nonumber \\&\quad + G_{pq}\frac{\partial ^2 u_p}{\partial x_q \partial z} +G^0 \frac{\partial ^2 w}{\partial z^2} \nonumber \\&\quad +A_1 \frac{\partial ^2 u_i}{\partial z \partial x_i}+(A_1+A_2) \frac{\partial ^2 w}{\partial z^2}=0, \end{aligned}$$

where $$i, k, p, q = 1, 2$$. Moreover,

5 $$\begin{aligned} K_{pqki}= & \mu \iint _{\Omega _A} \, \frac{\partial W_{Ak}^{pq}}{\partial X_i} \, \textrm{d}A+\iint _{\Omega _B} \, \frac{\partial W_{Bk}^{pq}}{\partial X_i} \, \textrm{d}A, \end{aligned}$$

6 $$\begin{aligned} K^0_{ik}= & \mu \iint _{\Omega _A} \, \frac{\partial W_{Ak}^{0}}{\partial X_i} \, \textrm{d}A+\iint _{\Omega _B} \, \frac{\partial W_{Bk}^{0}}{\partial X_i} \, \textrm{d}A, \end{aligned}$$

7 $$\begin{aligned} H_{pk}= & \mu \iint _{\Omega _A} \, \frac{\partial \phi _A^p}{\partial X_k} \, \textrm{d}A+\iint _{\Omega _B} \, \frac{\partial \phi _B^p}{\partial X_k} \, \textrm{d}A, \end{aligned}$$

8 $$\begin{aligned} G_{pq}= & \mu \alpha _A \iint _{\Omega _A} \, \frac{\partial W_{Ai}^{pq}}{\partial X_i} \, \textrm{d}A+\alpha _B\iint _{\Omega _B} \, \frac{\partial W_{Bi}^{pq}}{\partial X_i} \, \textrm{d}A, \end{aligned}$$

9 $$\begin{aligned} G^0= & \mu \alpha _A \iint _{\Omega _A} \, \frac{\partial W_{Ai}^{0}}{\partial X_i} \, \textrm{d}A+\alpha _B\iint _{\Omega _B} \, \frac{\partial W_{Bi}^{0}}{\partial X_i} \, \textrm{d}A, \end{aligned}$$

10 $$\begin{aligned} A_1= & (1+\alpha _A)\mu |\Omega _A|+(1+\alpha _B) |\Omega _B|, \end{aligned}$$

and

11 $$\begin{aligned} A_2= \mu |\Omega _A|+|\Omega _B|, \end{aligned}$$

where $\mu = \frac{\mu _A}{\mu _B}$ and $\alpha _{j} = \frac{\lambda _{j}}{\mu _{j}}$ for $j=A$ or *B*. Note that Eqs. (5–11) represent the effective homogenised Lamé parameters and areas over a single cell. Here $W_{ji}^{pq}(\textbf{X})$, $W_{j i}^{0}(\textbf{X})$ and $\phi _{j}^p(\textbf{X})$ (for $i, p, q = 1, 2$ and $j = A, B$) are microscale cell variables introduced to close the cell problem which must be solved over a single cell geometry with periodic boundary conditions on the external bounds of the cell geometry. To evaluate $W_{ji}^{pq}$, we solve

12 $$\begin{aligned} (1+\alpha _j) \frac{\partial ^2 W_{j i}^{pq}}{\partial X_k \partial X_i}+\frac{\partial ^2 W_{j k}^{pq}}{\partial X_i \partial X_i} =0 \end{aligned}$$

with boundary conditions on the interface, $\gamma$, between materials *A* and *B*, of continuity of displacement,

13 $$\begin{aligned} W_{Ak}^{pq} =W_{Bk}^{pq} \end{aligned}$$

and continuity of stress

14 $$\begin{aligned}&n_k \left( \mu \alpha _A \frac{\partial W_{Ai}^{pq}}{\partial X_i} -\alpha _B \frac{\partial W_{Bi}^{pq}}{\partial X_i}\right) +n_k(\mu \alpha _A-\alpha _B)\delta _{pq} \nonumber \\&\quad +\mu n_i \left( \frac{\partial W_{Ai}^{pq}}{\partial X_k}+\frac{\partial W_{Ak}^{pq}}{\partial X_i}\right) -n_i\left( \frac{\partial W_{Bi}^{pq}}{\partial X_k}+\frac{\partial W_{Bk}^{pq}}{\partial X_i}\right) \nonumber \\&\quad +n_i (\mu -1)(\delta _{ip} \delta _{kq}+\delta _{iq}\delta _{kp})=0. \end{aligned}$$

To evaluate $W_{ji}^{0}$, we solve

15 $$\begin{aligned} (1+\alpha _j) \frac{\partial ^2 W_{j i}^{0}}{\partial X_k \partial X_i}+\frac{\partial ^2 W_{jk}^{0}}{\partial X_i \partial X_i} =0 \end{aligned}$$

with interface boundary conditions on $\gamma$ of

16 $$\begin{aligned} W_{Ak}^0 = W_{Bk}^0, \end{aligned}$$

and

17 $$\begin{aligned}&n_k \left( \mu \alpha _A \frac{\partial W_{Ai}^0}{\partial X_i}-\alpha _B \frac{\partial W_{Bi}^0}{\partial X_i}\right) \nonumber \\&\quad +n_k (\mu \alpha _A -\alpha _B)+\mu n_i \left( \frac{\partial W_{Ai}^0}{\partial X_k}+\frac{\partial W_{Ak}^0}{\partial X_i}\right) \nonumber \\&\quad -n_i \left( \frac{\partial W_{Bi}^0}{\partial X_k}+\frac{\partial W_{Bk}^0}{\partial X_i}\right) =0 . \end{aligned}$$

To evaluate $$\phi _j^p$$,

18 $$\begin{aligned} \frac{\partial ^2 \phi _j^p}{\partial X_i \partial X_i} =0 \end{aligned}$$

is solved with interface boundary conditions on $\gamma$ of

19 $$\begin{aligned} \phi _A^p = \phi _B ^p \end{aligned}$$

and

20 $$\begin{aligned} n_i \left( \mu \frac{\partial \phi _A^p}{\partial X_i}-\frac{\partial \phi _B^p}{\partial X_i}\right) +n_p (\mu -1)=0. \end{aligned}$$

### Adapting homogenisation framework to peripheral nerve cross section

**Fig. 3:**
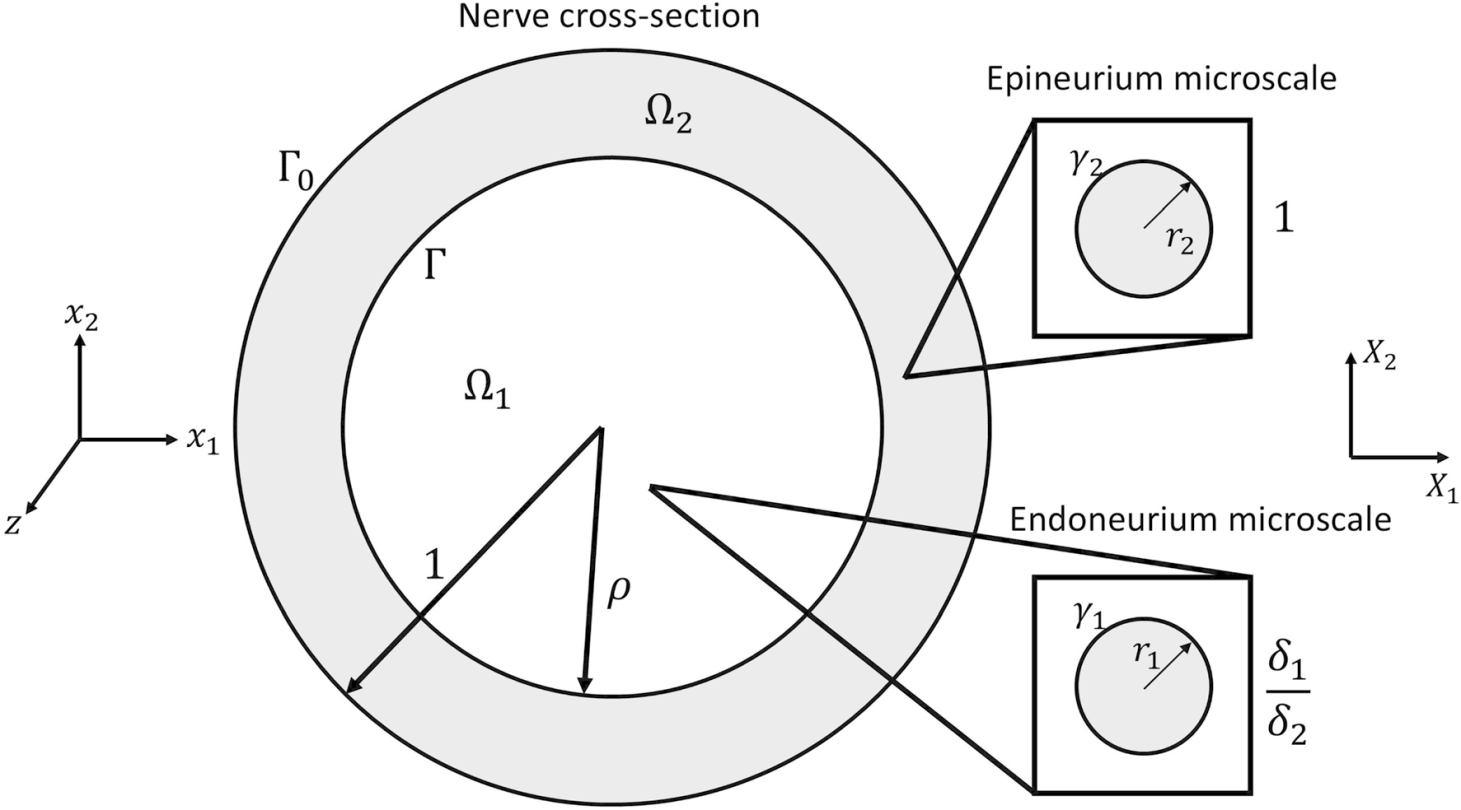
Non-dimensional lateral cross section of a simplified nerve containing a single circular fascicle, with radius $\rho$. Endoneurium area is $|\Omega _1|$ and epineurium area $|\Omega _2|$. Due to different fibril radii and number densities in the endoneurium and epineurium, separate microscales are introduced for the two different regions, but both are scaled by $\delta _2$, the microscale length scale in the epineurium

Section 2.1 develops the governing equations for the biomechanical behaviour a single fibre-supported composite material characterised by the length scales *d* and $\delta$, assuming small strains. We describe the endoneurium and epineurium as two different composite materials due to the observed differences in collagen fibril density seen in Mason and Phillips (2011), which we hypothesise leads to differing effective mechanical properties in the two regions. As shown in Fig. 3, we refer to these two different regions with subscript 1 denoting an endoneurial parameter and 2 denoting a epineurial parameter. To use the homogenisation method summarised in Sect. 2.1, we non-dimensionalise the macroscale cross section of the nerve relative to the diameter of the nerve, *d*, such that, for the case of a mono-fascicular nerve the fascicle will have a radius of $d \rho$ where $\rho$ is the non-dimensional fascicule radius where $0<\rho <1$. We denote the total non-dimensional area of the endoneurium as $|\Omega _1|$ and the area of the epineurium as $|\Omega _2|$. The outer boundary of the nerve cross section is denoted $\Gamma _0$, and the boundary between the endoneurium and epineurium is $\Gamma$. Within each region *i* (for $i=1$ or 2) we take the dimensional radius of the collagen fibrils to be $r^*_i$ and the number density per $\mu \text {m}^2$ of collagen fibrils to be $n_i$. We are then able to define an effective microscale length scale of each microscale unit within the endoneurium and epineurium as

21 $$\begin{aligned} \delta _i=\frac{1}{\sqrt{n_i}} \quad \text {for } i=1, 2. \end{aligned}$$

To ensure that the mechanical properties match across both the endoneurium and epineurium, we pick a single $\delta$ value across which we non-dimensionalise and use to define the relative small parameter $\epsilon$. The choice of this microscale length is arbitrary, as both $\delta$ values are assumed to be of similar orders, and thus here we non-dimensionalise using the collagen fibre scale within the epineurium, $\delta _2$. This leads to the non-dimensional microscale parameters used to formulate the homogenised problem, $r_1 = \delta _2 r_1^*$ and $r_2 = \delta _2 r_2^*$, with $\epsilon = \frac{\delta _2}{d}$.

The epineurium and endoneurium have distinct microscale variables, the number density and radius of fibrils, leading to differing homogenised equations of the form of Eqs. (3) and (4) in the two regions. The 3D displacements in both regions $\mathbf {u_i} = (u_i, v_i, w_i)^T$ for $i=1, 2$ can be obtained by solving these equations and the associated Cauchy stress tensors $\mathbf {\tau }_i$, as given in Appendix A. We assume the endoneurium and epineurium are fully bonded together on $\Gamma$ by applying continuity of displacement,

22 $$\begin{aligned} u_1 = u_2, \quad v_1 = v_2 \quad \text {and} \quad w_1=w_2, \end{aligned}$$

and continuity of normal stress,

23 $$\begin{aligned} \textbf{n} \cdot \mathbf {\tau }_1 = \textbf{n} \cdot \mathbf {\tau }_2, \end{aligned}$$

where $\textbf{n}$ is the normal to $\Gamma$ in the $(x_1, x_2)$-plane. Zero normal stress is also applied on $\Gamma _0$.

The epineurium and endoneurium have distinct microscale variables leading to differing homogenised equations of the form of Eqs. (3) and (4) in the two regions. We couple these two problem sets via boundary conditions on the interface between the two regions, where we impose continuity of displacements and normal stress. The overall longitudinal displacement of the nerve reduces to

24 $$\begin{aligned} w = {\left\{ \begin{array}{ll} w_1 \quad \text {within } \Omega _1, \\ w_2 \quad \text {within } \Omega _2, \end{array}\right. } \end{aligned}$$

and similarly for all other components of displacement and the Cauchy stress tensor. To evaluate the effective mechanical properties of the nerve we simulate a longitudinal tensile test of a section of nerve with constant lateral cross section. The maximum longitudinal non-dimensional length is denoted *L*. The base of the nerve is effectively fixed using the boundary conditions

25 $$\begin{aligned} u=v=w=0 \quad \text {at } z=0, \end{aligned}$$

and a lateral fixed displacement of *d* applied through

26 $$\begin{aligned} w = 1 \quad \text {at } z=L. \end{aligned}$$

Note, based on the assumption that the peripheral nerve is suitably long compared to the width of the nerve, the system decouples resulting in a plain strain problem. The effective axial Young’s modulus of the peripheral nerve is given by

27 $$\begin{aligned} E = \frac{\tau _{zz}}{\frac{\partial w}{\partial z}}, \end{aligned}$$

where $\tau _{zz}$ is the longitudinal stress component, given in Appendix A, and the effective axial Poisson ratio

28 $$\begin{aligned} \nu = - \frac{\frac{\partial u}{\partial x_1}}{\frac{\partial w}{\partial z}} = - \frac{\frac{\partial v}{\partial x_2}}{\frac{\partial w}{\partial z}}. \end{aligned}$$

### Investigating parameter variation

Due to the fundamental challenges of using data from a population of specimens, there is significant variation in the reported parameter values required for the model described in Sect. 2.2. In this subsection, we conduct a preliminary analysis to investigate which parameters are the most influential in determining effective mechanical properties within our model, and which have relatively little effect within the observed, or realistic, ranges.

**Table 1:** Table summarising microscale and geometric input parameters in two-layered model, summarised in Sect. 2.2 and using Eqs. (3) and (4), along with assumed values, parameter spaces explored and resultant Young’s modulus values, inferred using data values from Fung (1993), Phillips et al. (2004) and Mason and Phillips (2011)

Parameter	Notation	Value	Variation	Young’s modulus (GPa)
Lamé parameter of collagen fibril (GPa)	$\lambda _A$	0.4	0.04–0.5	0.817–0.880
Shear modulus of collagen fibril (GPa)	$\mu _A$	0.4	0.04–0.5	0.603–0.942
Lamé parameter of surrounding medium (GPa)	$\lambda _B$	0.1	0.04–0.5	0.847–0.960
Shear modulus of surrounding material (GPa)	$\mu _B$	0.36	0.04–0.5	0.407–1.064
Number of collagen fibrils per $\upmu m^2$ in endoneurium	$n_1$	218.5	200–300	0.868–0.891
Number of collagen fibrils per $\upmu m^2$ in epineurium	$n_2$	83.7	50–130	0.866–0.878
Diameter of collagen fibrils in endoneurium (nm)	$r^*_1$	42.5	30–60	0.849–0.921
Diameter of collagen fibrils in epineurium (nm)	$r^*_2$	77	60–90	0.867–0.877
Proportion of non-fascicular area	$\phi$	0.25	0.2–0.6	0.872–0.879

The microscale Lamé parameters of the collagen fibrils and the surrounding material within both the endoneurium and epineurium will impact upon the macroscale predictions our model generates using Eqs. (27) and (28). However, accurate measurement of these values is difficult due to the small size of these respective tissues. To infer the Lamé parameters we make the assumption that both materials are elastic and undergo small deformation within the normal range of motion exhibited by peripheral nerves. Furthermore, we make the simplification that both the collagen fibril and the surrounding material are compressible with Poisson ratios between 0 and 0.5; hence both $\lambda _j$ for $j=A, B$ are positive. We work within the model constraints that the non-dimensionalised constraints $\alpha _j$ (for $j = A, B$) and $\mu$ are of order unity, meaning both the collagen fibrils and the surrounding material have similar levels of rigidity. Graham et al. (2004) report a peak Young’s modulus value for a single collagen fibril of 32 MPa, while Wenger et al. (2007) report Young’s modulus values up to 11.5 GPa demonstrating the wide variation of values for the Young’s modulus of collagen fibrils present in the literature. For the purpose of this study, we will use the well accepted Young’s modulus value of 1 GPa for a single collagen fibril given in Fung (1993), as a starting point. Due to the lack of data on Poisson ratio of collagen fibrils we assume $\alpha _A=1$, to ensure the collagen fibril is compressible, with a Poisson ratio value of 0.5, leading to base values of $\lambda _A=\mu _A = 0.4$ GPa. We assume the material surrounding collagen fibrils will have lower Young’s and shear moduli than the collagen fibrils, hence we take $\lambda _B = 0.1$ GPa and $\mu _B = 0.36$ GPa, resulting in a Young’s moduli of 0.8 GPa for the surrounding material. We have chosen a parameter space of all Lamé parameters to ensure the non-dimensional constants in the model remain within the parameter space for which our model is valid. Finally, we used geometric data from Mason and Phillips (2011) to determine the base values and parameter spaces for all geometric parameters. These are summarised in Table 1.

**Fig. 4:**
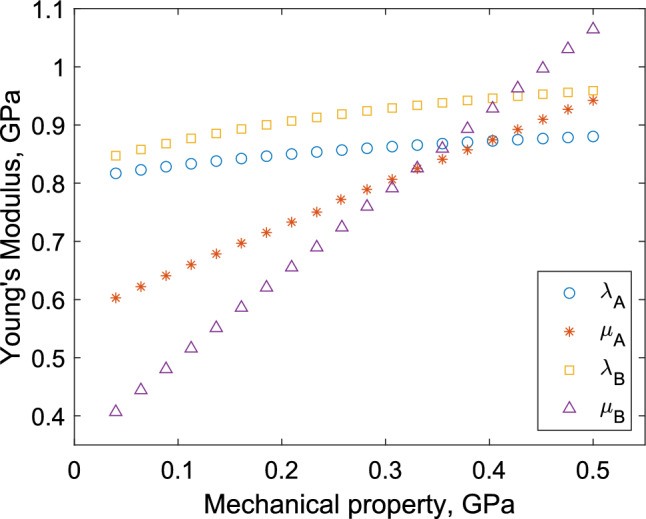
Effective Young’s modulus of a peripheral nerve, calculated using Eq. (27), when varying (i) $\lambda _A$, (ii) $\mu _A$, (iii) $\lambda _B$, and (iv) $\mu _B$, while holding all other parameters at the values stated in Table 1

In Fig. 4, we explore the impact of individually varying $\lambda _j$ and $\mu _j$, for $j = A, B$, on the effective axial Young’s modulus of the whole nerve. We see that the values of $\lambda _A$ and $\lambda _B$ have relatively little impact compared to the shear moduli. We have also examined variation in: (i) the number of collagen fibres, $n_1$ and $n_2$; (ii) the fibril diameters, $r_1$ and $r_2$; and (iii) proportion of non-fascicular area, $\phi$. Out of these geometric parameters varying the fibril diameters had the greatest effect but this was of the order of 0.07 GPa at the most. Comparing this to the variation shown in Fig. 4 we see that the key parameters that effect the Young’s Moduli, and so must be parameterised, are the micromechanical parameters, particularly the shear moduli, $\mu _A$ and $\mu _B$.

### Parameterising model

Here, we parameterise the micromechanical model parameters, $\lambda _A$, $\mu _A$, $\lambda _B$ and $\mu _B$, described in Sects. 2.1 and 2.2. To do this we require four mechanical measurements from rat peripheral nerves. For this purpose, we use the axial Young’s modulus value of $0.04096 \pm 0.000259$ GPa and axial Poisson ratio of $0.37 \pm 0.02$ given in Liu et al. (2012). To fully parameterise the model we clearly need two additional sets of mechanical data; however, data on the mechanics of peripheral nerves combined with macroscale and microscale geometrical data are scarce in the literature. To fill this gap, we consider bounds on the microscale mechanical properties $\lambda _A$, $\mu _A$, $\lambda _B$ and $\mu _B$. For the Lamé parameters we consider positive values of $\lambda _j$ and $\mu _j$ (for $j=A, B$) which lead to Poisson values in the endoneurium and epineurium between 0 and 0.5. This corresponds to the inequalities

29 $$\begin{aligned} 2 \mu _j< E_j < 3 \mu _j, \quad \text {for } j = A, \text { or } B. \end{aligned}$$

Additionally, we expect the epineurium, as the outer layer of a peripheral nerve, and so subject to greater bending forces, to be more flexible than the endoneurium and so we impose

30 $$\begin{aligned} E_B< E_A \quad \text {and} \quad \mu > 1. \end{aligned}$$

Conditions (30) do not easily translate to bounds on $\lambda _j$ and $\mu _j$, hence we introduce an additional variable *n* to translate the inequality in terms of $E_B$ into an inequality in terms of $\mu _B$, defined such that

31 $$\begin{aligned} E_B = n \mu _B. \end{aligned}$$

**Fig. 5:**
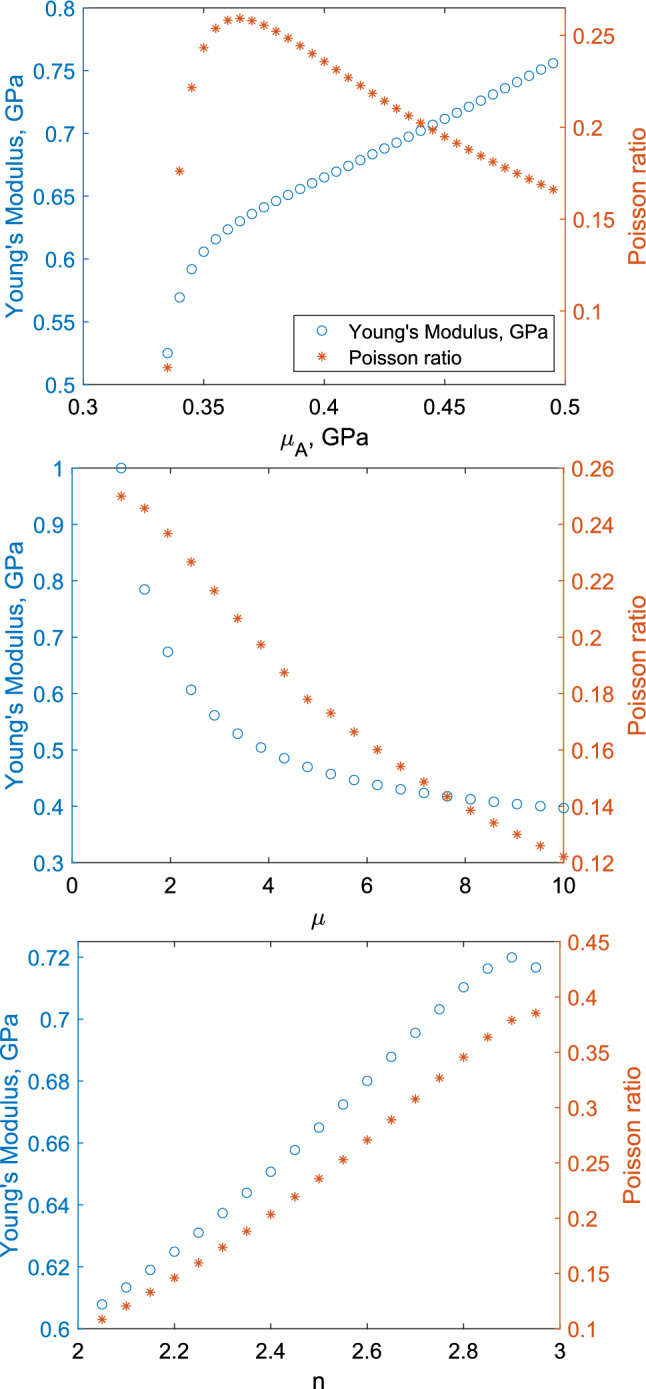
Effect of variation in $\mu _A$, $\mu$ and *n* on the predicted axial Young’s modulus and Poisson ratio. Values are fixed as $E_A=1$ GPa, $\mu _A=0.4$ GPa, $\mu =2$, $n=2.5$, $n_1=218.5$, $n_2=83.7$, $r_1=42.5$ $\upmu$m, $r_2=77$ $\upmu$m and proportion of non-fascicular area is 0.25, unless stated otherwise

**Fig. 6:**
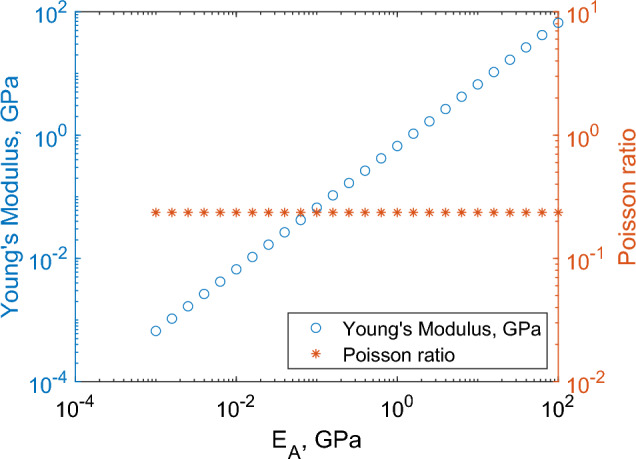
Effect of the magnitude of Young’s modulus of the collagen fibrils on the effective axial Young’s modulus and Poisson ratio. Values are fixed throughout: $\mu _A=0.4 E_A$ GPa, $\mu =2$, $n=2.5$, $n_1=218.5$, $n_2=83.7$, $r_1=42.5$, $r_2=77$ and the proportion of non-fascicular area is 0.25

In Fig. 5, we consider variability in $\mu _A$, $\mu$ and *n*, with the Young’s modulus of the collagen fibrils, $E_A$ equal to 1 GPa, (Fung 1993). We consider the regions

32 $$\begin{aligned} \frac{E_A}{3}< \mu _A< \frac{E_A}{2}, \quad \mu >1, \quad \text {and} \quad 2<n<3, \end{aligned}$$

based on assumptions (29) and (30). We then investigate the effect of $E_A$ on the effective axial Young’s modulus and the effective axial Poisson ratio, as shown in Fig. 6. We see that axial Poisson ratio is independent of $E_A$ and $\mu$, allowing us to fit $\mu _A$ and *n* using the value of the effective axial Poisson ratio and then systematically pick values of $E_A$ and $\mu$ to fit the value for effective axial Young’s modulus.

**Table 2:** Values for stiffness ratio between joint and non-joint regions for the sciatic and median nerve

Nerve	Stiffness ratio	
	Model estimate	Experimental data
Sciatic	0.96	$0.8 \pm 0.025$
Median	0.94	$0.525 \pm 0.075$

Experimental values are taken from Phillips et al. (2004)

**Table 3:** Range of possible stiffness ratio values as a result of varying microscale mechanical properties

	Maximum variation in			
	stiffness ratio due to:			
Nerve	$E_A$	$\mu _A$	$\mu$	*n*
Sciatic	0	0.006	0.0394	0.004
Median	$0.5 \times 10^{-4}$	0.155	0.0663	0.0059

Due to the limitations of data, we have been working with four unknowns and two mechanical data points to parameterise the model. To improve our estimates, we have compared our model to the values of stiffness ratio in Phillips et al. (2004), shown in Table 2. In Phillips et al. (2004), strain is measured *in situ*, while the limb is flexed for both the median and sciatic nerve in joint and non-joint regions. The stiffness ratio is calculated by taking the ratio between the gradients of the force-extensive curves in the joint and non-joint regions. We have replicated this calculation and displayed the results in Table 2. We see that we are able to predict a higher stiffness ratio in the sciatic nerve than the median nerve, consistent with experiments; however, we over predict the stiffness ratio in both nerves. We have run parameter sweeps to minimise this over-prediction, shown in Table 3, and we see different choices of the parameters $E_A$, $\mu _A$, $\mu$ and *n* cannot decrease the stiffness ratio to the degree seen in Phillips et al. (2004). We conclude we cannot find a mechanical regime that quantitatively matches the experimental results across both Liu et al. (2012) and Phillips et al. (2004). We have therefore chosen the mechanical regime which leads to an axial Young’s modulus of 0.0411 GPa and an axial Poisson ratio of 0.3651 for the joint region of the sciatic nerve. The full mechanical and structural setup leading to these values is summarised in Table 4, and we will use this micromechanical regime through the rest of this work.

**Table 4:** Base values for comparison with experimental work which lead to macroscale values of 0.0411 GPa for axial Young’s modulus and 0.3651 for axial Poisson ratio using data from Phillips et al. (2004) and Mason and Phillips (2011)

Variable	Base value	Variable	Base value
$\mu _A$	0.036 GPa	$\nu _A$	0.389
$\mu _B$	0.0045 GPa	$\nu _B$	0.35
$\lambda _A$	0.126 GPa	$n_1$	218.5
$\lambda _B$	0.0105 GPa	$n_2$	83.7
$\alpha _A$	3.5	$r_1$	42.5 nm
$\alpha _B$	2.33	$r_2$	77 nm
$\mu$	8	$\delta _1$	$6.765\times 10^{-8}$ m
$E_A$	0.1 GPa	$\delta _2$	$1.093\times 10^{-7}$ m
$E_B$	0.012 GPa	$\phi$	0.25
$\rho$	0.866		

## Results

Using the micromechanical regime representative of the rat peripheral nerve, summarised in Table 4, we will now consider the effect of (i) varying microstructural geometry through varying fibril radius and collagen proportion and (ii) varying macroscale geometry.

### Varying fibril radius and collagen proportion

Here, we consider whether our model confirms the experimental hypothesis that smaller fibril radii with a higher number density occur in more flexible regions of the nerve. To do this we consider variation from the mono-fascicular joint region of the sciatic nerve, for which the parameter regime is summarised in Table 4.

**Fig. 7:**
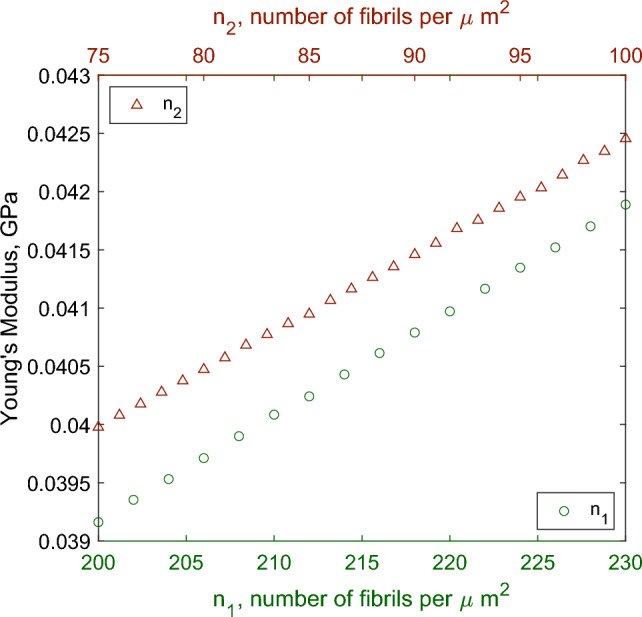
Effect on effective axial Young’s modulus of varying collagen fibril density in different regions when considering physical ranges and parameter values for the joint sciatic region

In Fig. 7, we consider the effect of varying the density of fibrils in the endoneurium and epineurium of the joint sciatic nerve. In both the endoneurium and epineurium, we see that an increasing number of fibrils results in a higher Young’s modulus for the whole nerve consistent with increasing the proportion of proportion of stiffer material in a composite material. To answer the question of whether this is an effect of the nerve microstructure or the collagen composition of the nerve, we repeat this simulation with firstly the amount of collagen in the nerve cross section held constant, and secondly, with total number of fibrils constant. The total non-dimensional amount of collagen within the endoneurium or epineurium in a cross section of a nerve is defined as

33 $$\begin{aligned} c_{1, 2} = \frac{r_{1, 2}^2 n_{1, 2} \pi }{4 \times 10^6}, \end{aligned}$$

where the total amount of collagen is

34 $$\begin{aligned} c = (1-\phi ) c_1 + \phi c_2. \end{aligned}$$

**Fig. 8:**
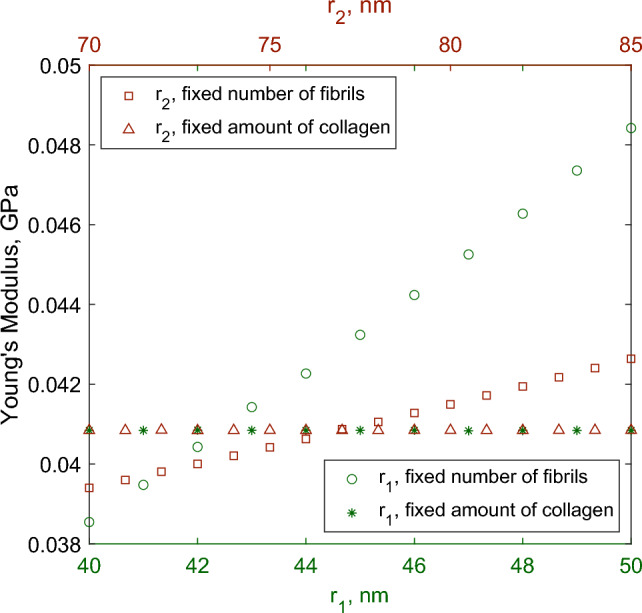
Effect on effective axial Young’s modulus of varying collagen fibril diameter in different regions when considering physical ranges and parameter values for the joint sciatic region. Total amount of collagen or total number of fibrils in each region are held constant

In Fig. 8, we see that when the number of fibrils is held to be constant but the amount of collagen increases with increasing fibril diameter, Young’s modulus increases as well. However, when the amount of collagen is held constant, we see that Young’s modulus is independent of any other parameter considered, showing here that it is the amount of collagen in a cross section of peripheral nerve at the microscale level that dictates the macroscale Young’s modulus.

**Table 5:** Non-dimensional amount of collagen in each region, $c_{1, 2}$, and total non-dimensional amount of collagen, *c*, for different nerves and regions

Nerve & region		$c_1$	$c_2$	*c*
Median	Joint	0.2736	0.4886	0.3618
	Non-joint	0.3145	0.4908	0.4000
Sciatic	Joint	0.3100	0.3898	0.3300
	Non-joint	0.3022	0.4298	0.3481

Calculated using mean values for fibril diameter, density and proportion of non-fascicular area

Table 5 shows the values for the non-dimensional amount of collagen in the two joint and non-joint regions of nerves, and the area-weighted average across both the endoneurium and epineurium calculated using data from Mason and Phillips (2011). We see that the amount of collagen in each of the endoneurium and epineurium individually and overall is larger in the non-joint region than that in the joint region with the exception of the sciatic endoneurium $c_1$ value. We note that there is a very small difference in the joint and non-joint sciatic values of $c_1$, which could easily be caused by a single outlier. Nevertheless, we see the finding that it is collagen content within the nerve cross section that determines the Young’s moduli of the overall nerve is consistent with this existing data.

### Varying cross-sectional geometry

We have shown that collagen content is the key microscale determining factor for axial Young’s modulus of a nerve. However, to reach this conclusion we assumed an axisymmetric nerve with a single fascicle. Here, we investigate how much this conclusion would change if we consider different nerve cross-sectional geometries. Namely we ask if our model is sensitive to changing the symmetry and fascicular proportions of the nerve.

**Fig. 9:**
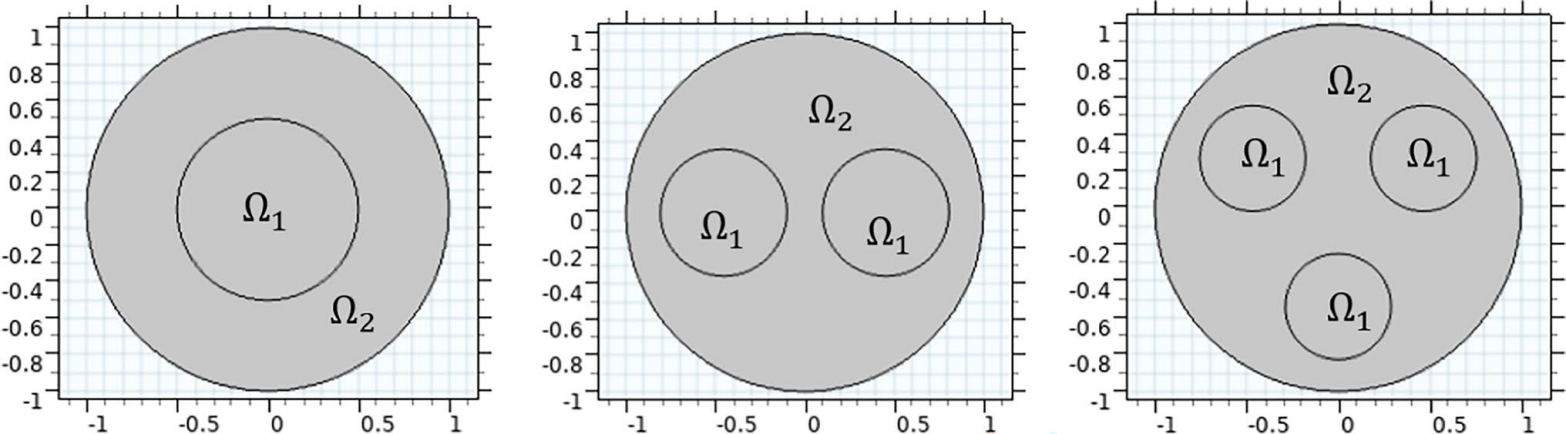
Macroscale geometries for one, two or three fascicles when preserving area of endoneurium. Proportion of non-fascicular area to total area is set to 0.75. Area of endoneurium is denoted $\Omega _1$ and epineurium $\Omega _2$ with parameters as given in Table 4

First, we examine if changing the number of fascicles from one to either two or three changes the axial Young’s modulus. The specific geometries used are shown in Fig. 9, and the effect of the different geometries on the Young’s modulus is summarised in Table 6. The proportion of non-fascicular area to total area, $\phi$, is set to either 0.75 or 0.9 to ensure symmetric, circular fascicles are possible. We observe that for all three arrangements, axial Young’s modulus is constant, with $\phi = 0.75$ resulting in an axial Young’s modulus of 0.045 GPa, and $\phi =0.9$ leading to an axial Young’s modulus value of 0.046 GPa. These values are rounded with some variation occurring at the fifth decimal place; however, these differences are small enough to be attributed to numerical noise and mesh variation as a result of the varying geometry. We conclude that the number of fascicles does not affect axial Young’s modulus when proportion of non-fascicular area is held constant.

**Table 6:** Effect of the total number of fascicles on Young’s modulus of the rat sciatic nerve, when total non-fascicular area is held constant

Number of fascicles	Young’s modulus (GPa)	
	$\phi = 0.75$	$\phi = 0.9$
1	0.04464	0.04600
2	0.04465	0.04600
3	0.04465	0.04600

Fascicles are circular and placed as in Fig. 9

**Fig. 10:**
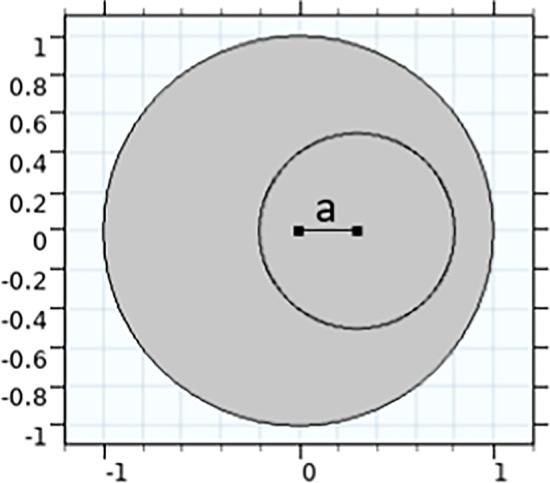
We define *a* to be the distance that the fascicle has been shifted off centre. In this figure, the proportion of non-fascicular area is set to be 0.75 to demonstrate the effect of *a*

**Fig. 11:**
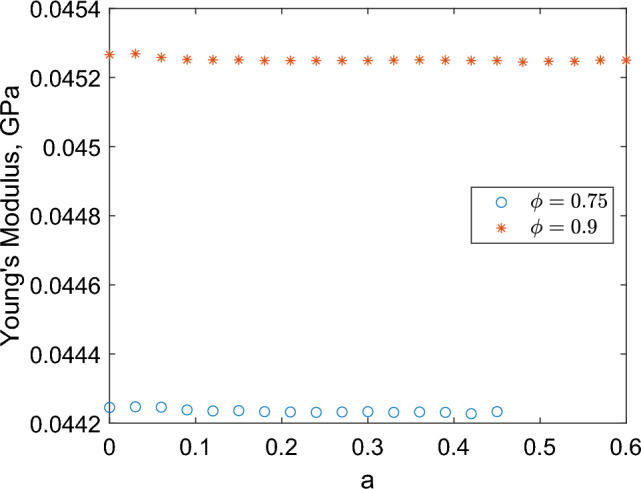
Effect on effective axial Young’s modulus when changing the asymmetry of the fascicle cross section. Here, *a* is the difference between the centre of the fascicle and the centre of the nerve, as shown in Fig. 10

**Fig. 12:**
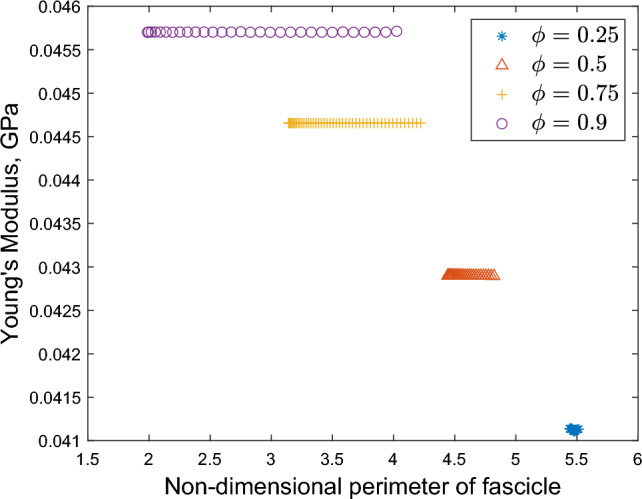
Effect of fascicle perimeter on effective axial Young’s modulus. Single fascicles are perturbed from circular to elliptical

Next, we consider the effect of changing the symmetry of the nerve cross section by perturbing the centre point of a single circular fascicle by a distance *a*, as shown in Figs. 10 and 11. Again we hold the proportion of fascicular area to non-fascicular area equal at either 0.75 or 0.9, and we consider the maximum range of possible values of *a*. We similarly see little variation in values with the rounded values being equivalent to those given for varying fascicle number.

Finally, we examine the effect of changing the shape of the fascicle, shown in Fig. 12. We hold the area of a single fascicle constant and perturb the area from a circle to an ellipse, calculating the axial Young’s modulus of the whole nerve. For all cases, we see no significant variation in the computed axial Young’s modulus as a result of variation in fascicle shape. When $\phi = 0.25$ we consistently observe an axial Young’s modulus of 0.041 GPa, and when increasing $\phi$ to 0.5 we observe a Young’s modulus of 0.043 GPa. In line with the analysis above, when $\phi = 0.75$ we observe an axial Young’s modulus of 0.045 GPa and similarly for $\phi = 0.9$ we observe an axial Young’s modulus value of 0.046 GPa. We conclude that compared to the proportion of fascicular area, the exact geometry of the nerve cross section has little appreciable variation on the macroscale mechanical properties of the overall nerve.

## Discussion

In this work, we have utilised a 3D multiscale biomechanical model for a peripheral nerve and parameterised it using mechanical and structural data on the joint region of a rat sciatic nerve. The hypothesis that thinner fibrils with higher density result in less stiff nerves was then investigated using the model. We saw that the model broadly agreed with this hypothesis but that this trend was principally due to the collagen content in the cross section of a nerve, rather than microscale fibular structure. The experimental data were re-analysed, and we saw that there was less collagen in less stiff regions in the original experimental data, supporting the biomechanical modelling conclusion. Finally, we examined whether or not changes in the macrostructure of the nerve would change the model outcomes and we saw that our results are independent of the macroscale cross section.

These results have implications for what experimental data are needed to be recorded to infer mechanical properties. In the literature, measures such as shape and perimeter of fascicules have previously been included, which although can be determined using software such as ImageJ or Fiji, much more manual input is required compared to evaluating areas or number of regions. Here, we have seen that the main measurements required to infer the Young’s modulus of a nerve can be summarised as:

1. Area of cross section of nerve;
2. Ratio of endoneurium to epineurium areas within a cross section of nerve;
3. Proportion of endoneurium containing collagen fibrils; and similarly,
4. Proportion of epineurium containing collagen fibrils.

In addition, for the full model described in this paper to be used in the future studies, four measurements of the mechanical properties, such as Young’s modulus or Poisson ratio, of multiple distinct samples should be taken and paired with the relevant the structural data from the same samples to parameterise the micromechanical properties $\lambda _j$ and $\mu _j$, (for $j = A$, or *B*). One of the main difficulties of this work has been working with structural and mechanical data from multiple sources and taking both sets of measurements from the same samples or sample sets would have resulted in the far more physiologically accurate models needed for the future development of engineered neural tissue. This paper uses the Young’s modulus value for a single collagen fibril of 1 GPa, but the modelling framework remains applicable over a range of Young’s modulus values. Further mechanical tests on tissue samples combined with the multiscale modelling framework described within this paper could provide a route to refine the range of mechanical properties of collagen fibrils.

This work also suggests that since it is the area of collagen fibrils that dictates elastic mechanical properties, we may use the much simpler area- or volume-averaging models to infer elastic mechanical properties rather than the complex multiscale biomechanical model described in this paper. The principal caveat for this conclusion is that one of the key assumptions within our model was that both the collagen and the surrounding material are linearly elastic. This simplification was necessary to focus on the effect of the multiple scales on the nerve biomechanics, within the limitations of current data, and to enable the comparison to Young’s modulus values within the literature, which assumes linear behaviour. Should future studies wish to incorporate more complex mechanical measures, such as bending modulus or yield point, which describe the material response to behaviours such as buckling or twisting, more complex models of the form presented in this paper will be required. Future models should explore the effects of nonlinear, fluid, viscoelastic or plastic regimes on the different components of peripheral nerves. However, with these more complex models, additional mechanical data will be required for model parametrisation and it will be imperative to conduct a more extensive experimental exploration of mechanical behaviour alongside biomechanical modelling efforts.

## Data Availability

Not applicable.

## Appendix A Stress components for a fibre-supported slender material

For a fibre-supported slender material consisting of two materials, A and B, we may write the stress tensor as

A1 $$\begin{aligned} \tau = \begin{pmatrix} \tau _{xx} & \quad \tau _{xy} & \quad \tau _{xz} \\ \tau _{xy} & \quad \tau _{yy} & \quad \tau _{yz} \\ \tau _{xz} & \quad \tau _{yz} & \quad \tau _{zz} \end{pmatrix}, \end{aligned}$$

wherein the individual components are given, as in Doman et al. (2022), using Eqs. (5–11):

A2 $$\begin{aligned} \tau _{xx}&= (G_{11}+A_1+A_2+2K_{1111}) \frac{\partial u}{\partial x} \nonumber \\&\quad +(G_{12} +2K_{1211}) \frac{\partial u}{\partial y} +(G_{21}+2K_{2111})\frac{\partial v}{\partial x}\nonumber \\&\quad +(A_1-A_2+G_{22}+2K_{2211})\frac{\partial v}{\partial y}\nonumber \\&\quad +(A_1-A_2+G^0+2K^0_{11})\frac{\partial w}{\partial z}, \end{aligned}$$

A3 $$\begin{aligned} \tau _{xy}&= (K_{1112}+K_{1121})\frac{\partial u}{\partial x} + (K_{1212}+K_{1221}+A_2) \frac{\partial u}{\partial y} \nonumber \\&\quad +(K_{2112}+K_{2121}+A_2)\frac{\partial v}{\partial x} +(K_{2212}+K_{2221})\frac{\partial v}{\partial y} \nonumber \\&\quad + (K_{12}^0+K_{21}^0) \frac{\partial w}{\partial z}, \end{aligned}$$

A4 $$\begin{aligned} \tau _{yy}&= (G_{11}+A_1-A_2+2K_{1122})\frac{\partial u}{\partial x} \nonumber \\&\quad +(G_{12}+2K_{1222})\frac{\partial u}{\partial y}+(G_{21}+2K_{2122})\frac{\partial v}{\partial x} \nonumber \\&\quad +(G_{22}+A_1+A_2+2K_{2222})\frac{\partial v}{\partial y}\nonumber \\&\quad + (G^0+A_1-A_2+2K_{22}^0)\frac{\partial w}{\partial z}, \end{aligned}$$

A5 $$\begin{aligned} \tau _{xz}&= (H_{11}+A_2)\left( \frac{\partial u}{\partial z}+\frac{\partial w}{\partial x}\right) \nonumber \\&\quad +H_{21}\left( \frac{\partial v}{\partial z}+\frac{\partial w}{\partial y}\right) , \end{aligned}$$

A6 $$\begin{aligned} \tau _{yz}&= H_{12}\left( \frac{\partial w}{\partial x}+\frac{\partial u}{\partial z}\right) \nonumber \\&\quad + (H_{22}+A_2)\left( \frac{\partial w}{\partial y}+\frac{\partial v}{\partial z}\right) \end{aligned}$$

and

A7 $$\begin{aligned} \tau _{zz}&= (G_{11}+A_1-A_2)\frac{\partial u}{\partial x} +G_{21} \frac{\partial v}{\partial x} \nonumber \\&\quad +G_{12}\frac{\partial u}{\partial y} +(G_{22}+A_1-A_2)\frac{\partial v}{\partial y} \nonumber \\&\quad + (G^0+A_1+A_2)\frac{\partial w}{\partial z}. \end{aligned}$$

## Code availability

Code available at https://github.com/eleanordoman/peripheral_nerve_paper_code.
